# Supplementary figures and images for: VIRify: An integrated detection, annotation and taxonomic classification pipeline using virus-specific protein profile hidden Markov models
Source: PLoS Comput Biol. 2023 Aug 28;19(8):e1011422. doi: 10.1371/journal.pcbi.1011422 (PMC10491390; doi:10.1371/journal.pcbi.1011422)

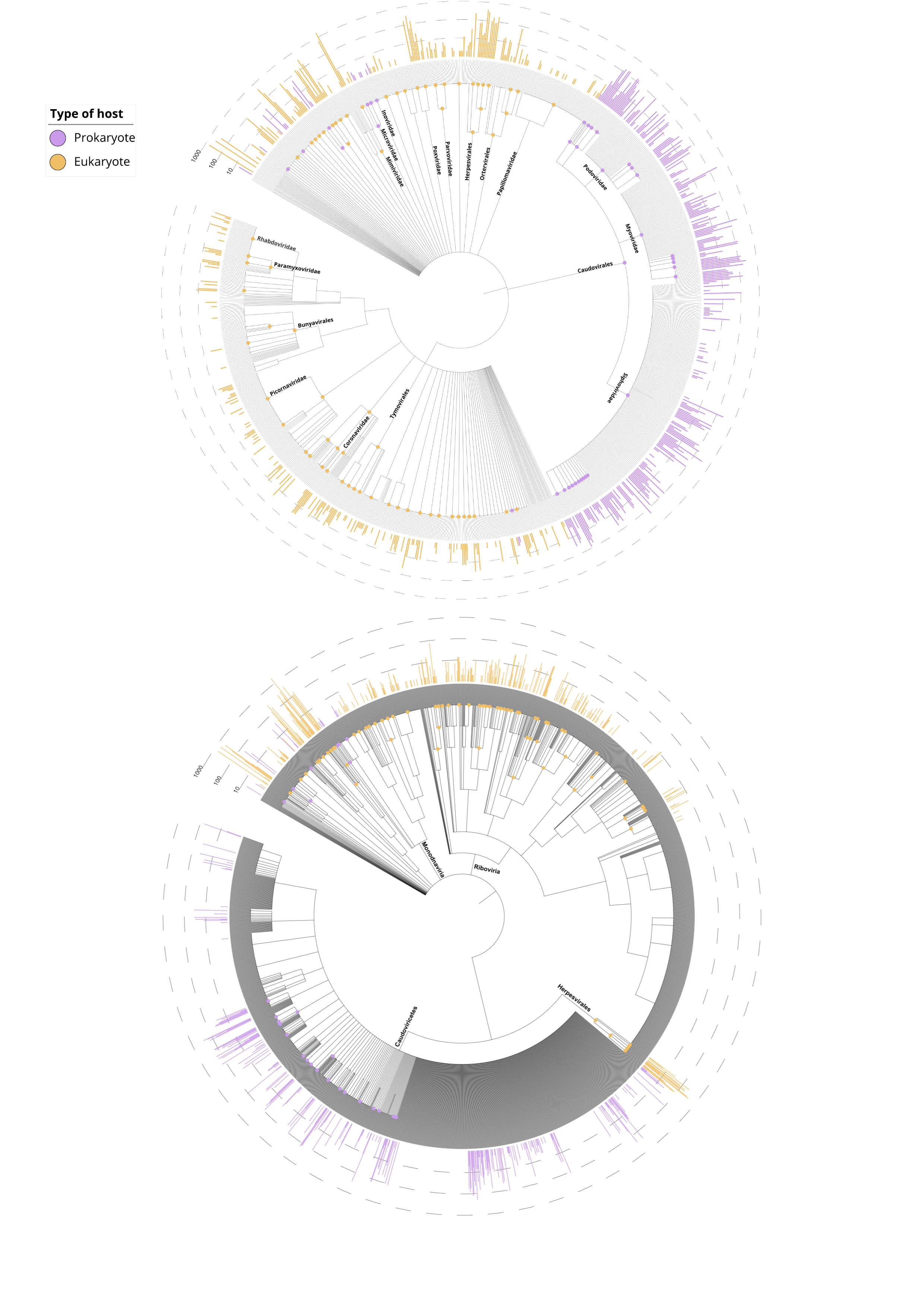

Supplement: S1 Fig — Circular dendrograms showing the NCBI virus taxonomy from March 2020 (upper panel) and January 2023 (lower pannel), with taxa covered by informative ViPhOGs highlighted with either dots or bars, and using the same colour scheme as in Fig 2. For the taxonomic ranks (order, family and subfamily) dots were used to indicate whether any informative ViPhOGs were identified for the corresponding taxon. By contrast, bars were used for taxa in the genus rank (tree’s leaves) to indicate the number of different informative ViPhOGs identified for the corresponding genus, as indicated by the numeric scale in the outer rings. (PNG) [file pcbi.1011422.s001.png]

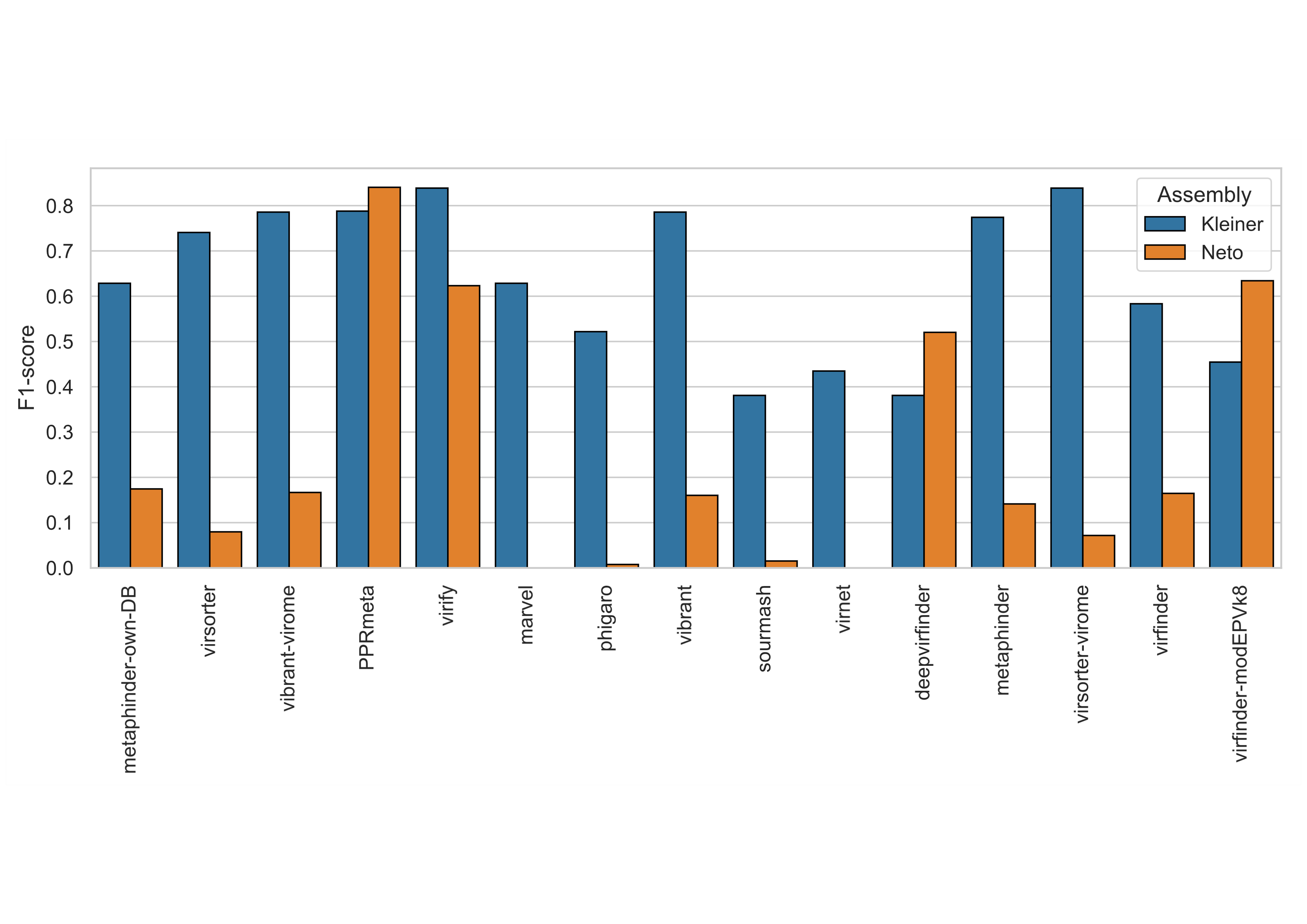

Supplement: S2 Fig — (PNG) [file pcbi.1011422.s002.png]

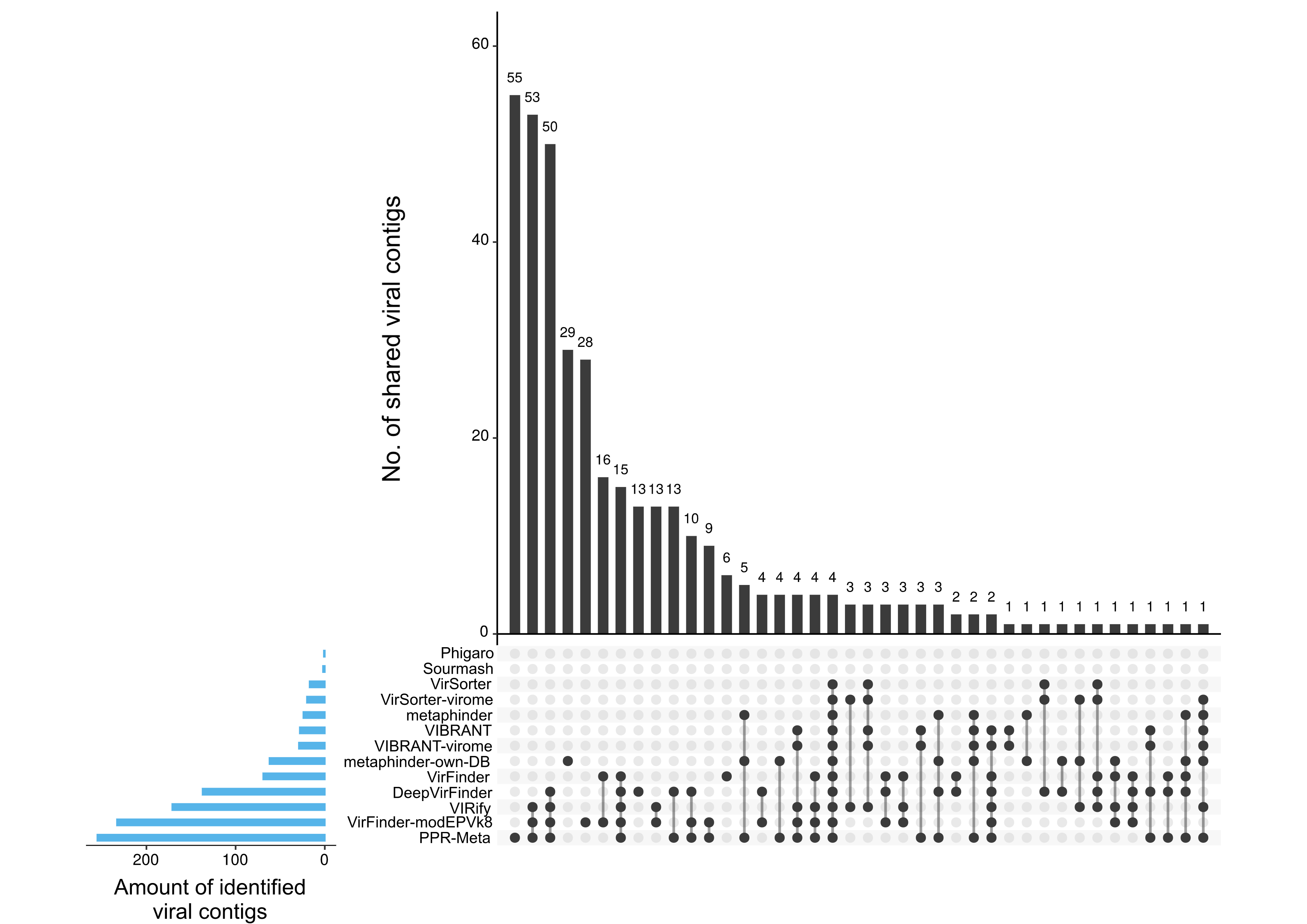

Supplement: S3 Fig — Viral prediction tools are compared and overlapping sets are shown. (PNG) [file pcbi.1011422.s003.png]
