# Supplementary material for: VIRify: An integrated detection, annotation and taxonomic classification pipeline using virus-specific protein profile hidden Markov models
Source: PLoS Comput Biol. 2023 Aug 28;19(8):e1011422. doi: 10.1371/journal.pcbi.1011422 (PMC10491390; doi:10.1371/journal.pcbi.1011422)
Supplement: S4 Fig — Combined results for high confidence, low confidence, and putative prophage hits are shown. VIRify was run in v0.2 and with the—virome option and a contig length filtering of 5000 nt. (PDF) [file pcbi.1011422.s004.pdf]

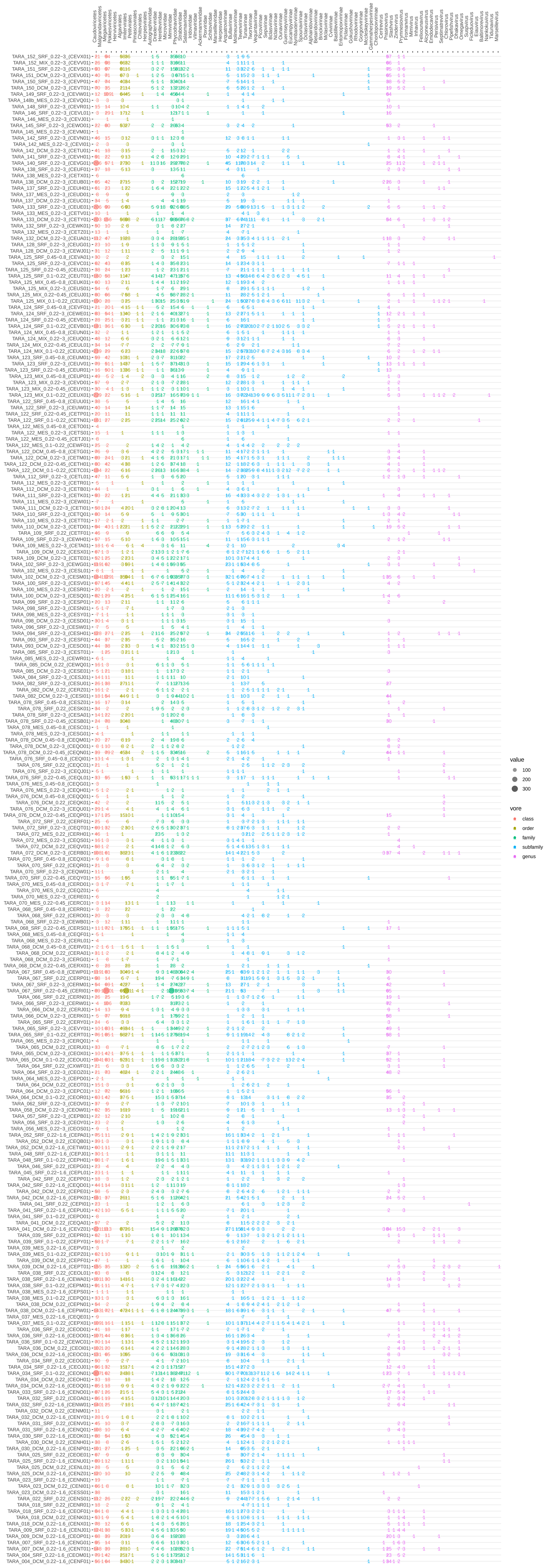
value
100
200
300
vore
class
order
family
subfamily
genus
