# Supplementary material for: VIRify: An integrated detection, annotation and taxonomic classification pipeline using virus-specific protein profile hidden Markov models
Source: PLoS Comput Biol. 2023 Aug 28;19(8):e1011422. doi: 10.1371/journal.pcbi.1011422 (PMC10491390; doi:10.1371/journal.pcbi.1011422)
Supplement: Attachment — Submitted filename: Response_to_reviewers.pdf [file pcbi.1011422.s005.pdf]

Response letter

We thank the reviewers for thoughtfully reading our manuscript and for their constructive comments, which we address below. We took the helpful comments as a motivation to also revise the pipeline code and update the taxonomic annotation and parameters to improve VIRify's predictions. We also added an additional data set to test the new version of VIRify for taxonomically classifying viral genomic sequences from previously published human gut metagenomes.

—

Reviewer #1
The authors have addressed a large issue within the field, although issues remain with annotation, VIRify clearly competes well with other tools in the field.

The fact that the authors have used two mock communities is great, although I do find the fact that comintamination within them is noted as a potential issue, troubling. Clearly better mocks are required in future studies, but I believe that to beyond the scope of this paper.

Thanks for your review. Yes, we agree that good "gold standard" test data sets are crucial, and by using the selected mock communities we tried to achieve some comparability. However, this is still an open problem beyond the development of VIRify, especially in light of all the different viruses spanning from dsDNA to ssRNA.

Major issues;
- The basis for the HMMs is the viral genomes from 2015, this is 7 years ago, meaning alot of diversity is missing. Is this something that can be easily updated? If not, this poses a major limitation which the authors should address in the text of the paper.

We agree that the set of genomes used to build the current collection of ViPhOGs does not include all viral genomes currently available in the NCBI databases. To get further insights into this issue we determined the coverage of the viral taxonomy available at NCBI in January 2023. A comparison of this with the coverage of NCBI's viral taxonomy from March 2020 demonstrates that the percentage of covered taxa for all assessed taxonomic ranks was indeed reduced.

As you have already suspected, updating the ViPhOGs to include all the viral genomes currently available in the NCBI databases and selecting the ones that could be used as markers for the currently known viral taxa is possible, but is out of the scope of this manuscript. We wanted to demonstrate primarily that our curated set of ViPhOGs and their corresponding hmmscan parameters could serve as the basis for a taxonomic classification approach for viral genomic sequences. Our results show that the taxonomic classification method developed here works very well for viral taxa covered by the informative ViPhOGs. However, to prevent the reporting of taxa that no longer exist in the viral taxonomy, we again updated the NCBI taxonomy file used by the pipeline to the version published on January 2023. We also eliminated all informative ViPhOGs that had been selected as markers for taxa that were not present in the updated taxonomy file anymore. By that, we could rerun the pipeline and update all results presented in our manuscript.We consider that an appropriate update that should

take care of recent changes in viral taxonomy. Updating the ViPhOGs to include newly available genomes and identifying markers for novel taxons will be the aim of future work but is out of the scope of the current work.

- I see no justification for the 10% ViPhOG annotation cutoff or that at least 2 hits are reported at a set level with 60% consistency. Please benchmark these factors to define why these thresholds are suitable.

We agree that these selected cut-offs needed more comprehensive benchmarking to make them more robust and to justify their selection as default parameters. In this context, please also note that the user can easily adjust them via the input parameters --prop and --taxthres. So far, we set these parameters to the described default values based on our benchmarking and test runs during the development of the pipeline. Now, and as described in the revised version of the manuscript (lines 250 - 265), we decided to replace these cutoffs with specific thresholds for each taxon. These were determined based on the number of corresponding informative ViPhOGs and the average number of CDS present in the complete genomes that each taxon covers from the entries available in the NCBI databases. Furthermore, we implemented an additional control step that checks whether the number of predicted CDS in a contig exceeds the average number of CDS times 2 standard deviations for the assigned taxon (lines 408 - 410). This step is useful to identify contigs whose size is significantly larger than the average size of the viral genomes covered by the assigned taxa, thus eliminating potential false positive assignments. These changes led to notable improvements in the classification of viral contigs from the mock datasets, particularly for the Neto co-assembly.

—

Reviewer #2
Rangel-Pineros et al are describing a pipeline "VIRify" that can detect viral contigs in a metagenomic assembly and perform taxonomy annotations using protein-similarity. The pipeline takes a pre-assembled set of contigs as an input and classifies them using a combination of several viral-detection software tools. Contigs classified as viral are further subjected to the taxonomy characterization: protein-coding ORFs are predicted for each contig and these ORFs are matched against a set of protein domain models (informative ViPhOGs) representative for different viral genera, families or orders. Authors validate their choice of software packages for each step of the pipeline and then benchmark it against 2 metagenomic datasets with experimentally known combinations of viruses/phages. Selection of protein-domain models (informative ViPhOGs) is briefly described in the manuscript but has been previously published elsewhere.

From the software perspective, "VIRify" adheres to the best practices: the pipeline is implemented using 2(!) Workflow management systems (nextflow and CWL), software packages VIRIfy relies on are containerized using docker/singularity for portability, documentations is thorough and contributors appear active on the github page of the repository.

The manuscript itself is well written and easy to follow for the most part, although it could be shortened somewhat by skipping redundant descriptions and unnecessary details in the introduction (see below).

Thanks for your review and for also acknowledging our implementation efforts. In this context, while we started to implement VIRIfy using two Workflow Management Systems (CWL and Nextflow), we decided now to only maintain Nextflow in the future. We see Nextflow as a more robust and more widely used WMS. We changed the manuscript text accordingly to focus on Nextflow.

I do have several comments that could help improve the manuscript:

Major:

1. Elaborate more on the significance of the “TARA Oceans" analysis - right now it is unclear if ViRIfy provides novel insight into those datasets or if ViRIfy is valuable as a convenient tool/utility here ? Can “Massive expansion of human gut bacteriophage diversity” paper by some of the co-authors be used to strengthen the utility of VIRify’s approach ?

Thanks for your suggestions. We have expanded our discussion of the TARA Oceans datasets and included new text both in the results and discussion sections about the use of VIRify for classifying human gut viral genomes from the Gut Phage Database (lines 780 - 814, and 956 - 969).

2. Reading the “Selection and comparison of virus prediction tools” section in Methods 239-286, the choice of 3 viral detection software seems somewhat arbitrary - i.e. according to SFig2 PPR-Meta outperformed most of the other tools - why not use it alone ? Figure 3 helps answer that a bit, but then maybe there is another way to justify the choice of VirSorter, VirFinder, PPR-Meta ?

We agree that based on the results presented in SFig2 it would be reasonable to select only PPR-Meta for detecting the presence of viral contigs in the input metagenomic assemblies, especially considering the benchmarking results obtained for the Neto assembly. However, we decided to also include VirSorter and VirFinder-modEPVk8 in our viral detection step largely for the following reasons. Even though PPR-Meta performs very well in detecting viral sequences (and especially in distinguishing them from microbial sequences, Wu *et al.* 2023, https://doi.org/10.1101/2023.04.26.538077), the underlying model was not explicitly trained to also detect prophages in bacterial contigs. Our aim was to design a pipeline capable of characterizing the viral fraction of a metagenomic assembly as comprehensively as possible, hence the importance of developing an approach suitable for the detection of both free viruses and prophages. VirSorter was designed for detecting both free phages and prophages, and many studies have successfully employed this tool to detect prophages in genomes sequences from a diverse range of bacterial lineages. Our results provide a few instances of prophages that were successfully detected by VirSorter, but missed by PPR-Meta (Figure 3).

Another reason why we decided to use the combination of viral predictors instead of just PPR-Meta is that despite its high sensitivity, this tool tends to call a relatively higher number of false positives in comparison with other tools (see also Ho *et al*. 2023, https://doi.org/10.1186/s40168-023-01533-x). On the other hand, our results show that the

use of this tool vastly improves the detection of viral sequences in assemblies from communities largely composed of eukaryotic viruses. Therefore, to support the detection of sequences from eukaryotic viruses but limit the number of false positive viral predictions, we decided to combine the predictions reported by PPR-Meta with the ones reported by VirFinder. We modified the manuscript's text accordingly to improve the explanation of the rationale for using the combination of tools instead of just PPR-Meta (lines 886 - 898).

Minor:
1. Shortening of the manuscript would improve readability, here are several examples:
a. 84-90 lytics vs lysogenic life cycles of phages - interesting - but in my opinion distracting
b. Shorten ViPhOG related description that were covered in the previous publication - e.g. in the first section of "Results"

Thank you so much for your suggestions. We modified the text accordingly and condensed it to improve its readability.

2.Provide references to supplementary figures in "Selection and comparison of virus prediction tools" section

Thanks for pointing this out. We agree that referencing the supplementary figures in the mentioned section would help support the claims stated therein. We have referenced all the relevant figures in the "Selection and comparison of virus prediction tools" section.

3.It would be nice to include a paragraph on ViRify's runtime expectations - i.e. how long it would run on a "typical" dataset, what hardware is recommended

Thanks for the comment, we added a short paragraph about runtime expectations and different hardware specifications to the manuscript (lines 1041 - 1062). We agree, that it is interesting for the user to get a ballpark idea of how long the pipeline takes. Due to the various steps, comprising viral predictions and especially the hmmscan commands, the pipeline can actually run quite long on limited hardware and that's why we recommend running VIRify on an HPC or the Cloud for larger data sets. While the pipeline runs reasonably fast on the two mock community data sets on a decent laptop (with 8 cores ~16 minutes for Kleiner co-assembly and ~30 minutes for Neto co-assembly), the 243 TARA Ocean assemblies need 2 days 13 hours on an HPC with SLURM and using the default configuration profile for cluster execution with pre-configured resources for each process (without the time needed for downloading all databases once, but also including fluctuation and pending jobs because the HPC is used by many). Experienced users can tweak the resources (CPU, RAM) in the Nextflow configuration if more are available on an HPC or the cloud.

4.Several of the tools that VIRify depends on are deep learning based - do they require GPUs or CPU is sufficient? Do you take it into account for containerization ?

VIRify can be run without GPUs. However, you are right, that, e.g., PPR-Meta is Deep Learning-based but the tool does not require any GPUs and runs reasonably fast on CPUs (anyway faster than, e.g., VirSorter or VirFinder). While it would be possible to add GPU support to the Nextflow pipeline, e.g., by adding a corresponding config file for the processes that support GPUs, we did not see any necessity to do that at the moment. However, when it

becomes relevant, that can be configured in the framework of Nextflow. Regarding the containers: Nextflow allows you to define architecture-specific flags to enable GPU support and mount container images accordingly. For example, one could add a flag to run specific pipeline processes (such as PPR-Meta) with GPU support on an HPC with SLURM. However, as said above, we do not do that now but could implement it for future tools that take huge advantage of GPU execution.

5. Could you discuss an alternative approach of taxonomy classification based on “complete” viral genomes (e.g. as in “Phanta: Phage-inclusive profiling of human gut metagenomes” preprint by Pinto et al) - what are some advantages/limitations relative to protein-similarity based approach ?

Thanks for your suggestion. We have expanded the manuscript’s discussion to include text commenting on the use of taxonomic classification approaches based on “complete” viral genomes and how they compare to protein-similarity based methods (lines 1001 - 1019).

—

Reviewer #3:
The manuscript by Rangel-Pineros and colleagues describes the creation of a new pipeline for virome analyses called VIRify. This pipeline is avalaible from GitHub as a Nextflow pipeline or CWL implementation. In brief, the pipeline uses either reads or assemblies, predicts which contigs are viral and provides a taxonomic classification using virus-specific protein profile HMMs derived from a separate study. The pipeline was tested on two virome mock communities and a TARA Oceans metagenomics study. The development included an analysis of the appropriateness of several different virus prediction tools using the “What the Phage” workflow (Marquet et al, 2022).

VIRify has a great strength that makes it stand out from other pipelines, the use of the ViPhOGs, a well-curated set of 22,013 orthologous protein domains that are informative for virus taxonomy. Using this approach, viral contigs can be rapidly classified against the taxonomy associated with the ViPhOGs, regardless of the type of virus (eukaryotic, bacterial, archaeal) or genome type (DNA/RNA), as long as there are representative ViPhOGs in the database.

Thanks for your review and for highlighting one of the key strengths of our pipeline compared to other publicly methods available, as well as our efforts in generating the curated set of informative ViPhOGs.

This leads me into my main comment on the manuscript and pipeline, the taxonomy itself is outdated and wrong. On lines 209-2216, the authors write that the Virus-Host DB was used in November 2019 and the NCBI Taxonomy in March 2020. By the time this paper is published, the taxonomy will be at least four years out of date. The NCBI Taxonomy in March 2020 still used the taxonomy release issued by the International Committee on Taxonomy of Viruses in 2019, Master Species List MSL#34 (https://ictv.global/taxonomy/history) which contains 14 orders, 150 families, 79 subfamilies, 1019 genera and 5560 species. In contrast, the current taxonomy release (MSL#37) comprises additional higher order taxa: 6 realms, 10 kingdoms, 17 phyla, 39 classes, 65 orders, 233 families, 2606 genera and 10434 species (Walker et al,

2022, table 1, https://link.springer.com/article/10.1007/s00705-022-05516-5/tables/1). Importantly, this taxonomy release saw the removal of the bacteriophage families Myoviridae, Podoviridae and Siphoviridae which were not monophyletic, and the removal of the order Caudovirales in favour of the class Caudoviricetes, the taxa most commonly recovered from metagenome studies (see Fig 3 and 5 for example). In addition, many new families of archaeal viruses have been established since 2019, and entirely new phyla have been defined with viruses that had never before been classified.

Since virus taxonomy is dynamic when new sets of viruses are discovered, any pipeline that claims to provide a taxonomic classification of viruses needs to be able to incorporate yearly updates to its taxonomy, or make clear disclaimers that the taxonomic classification is not up-to-date. In the case of VIRify, I presume updating the taxonomy could be achieved in a relatively straightforward way by remapping the ViPhOG database to the latest taxonomy database.

Thank you for your comment and for highlighting this issue regarding the status of the viral taxonomy underlying our pipeline. We are aware of the many changes that the viral taxonomy has undergone during the time span you mentioned. As a result, we have undertaken the following steps to address this issue.

First, we checked whether the taxa associated with the informative ViPhOGs were present or absent in the ICTV's MSL#38. We determined that only 29 out of the 616 taxa associated with the informative ViPhOGs were no longer present in the viral taxonomy, and as you pointed out, these included the families *Myoviridae*, *Siphoviridae,* and *Podoviridae*, and the order *Caudovirales*. Therefore, all the corresponding informative ViPhOGs were excluded from the original set, resulting in a reduction from 22,013 to 20,266 HMMs (~8%). Second, we updated the NCBI taxonomy file used for the taxonomic assignment step to provide up-to-date viral lineages that allow the pipeline to report updated taxonomic classifications after applying the implemented LCA approach. We also modified the pipeline's scripts to include the Class rank both in the tabular and graphical output.

As you indicated in your comment, the number of taxa in the viral taxonomy has increased dramatically since March 2020. We calculated the extent to which the informative ViPhOGs cover the January 2023 release of NCBI's viral taxonomy and compared it with the coverage we had previously determined for the March 2020 release. As expected, there was a noticeable reduction of coverage at all the examined ranks and we updated the results in the manuscript to reflect and discuss these changes. According to the results we obtained with the mock community assemblies, the current set of informative ViPhOGs performs very well for the taxonomic classification of contigs from viruses associated with the taxa known when the HMMs were generated. These observations demonstrate that our curated set of informative ViPhOGs is a powerful resource for performing taxonomic classifications of viral sequences from various lineages. These include many widespread genera and subfamilies within the *Caudoviricetes*, and many notable groups of eukaryotic viruses such as the coronaviruses and herpesviruses. However, we agree with you that covering a larger extent of the viral taxonomy is imperative to provide the community with a robust resource that generates comprehensive taxonomic profiles of viral communities.

For the next iteration of VIRify, a new set of informative ViPhOGs that covers the complete viral genomes currently available in the public databases will be generated. As you suggested, keeping the ViPhOGs' taxonomic associations updated can be achieved by remapping the HMMs to the latest taxonomy database. However, if a taxon is eliminated or its definition is drastically changed, then the corresponding informative ViPhOGs might need to be re-generated, which was beyond the scope of this manuscript. Nonetheless, keeping the ViPhOGs up-to-date with the viral taxonomy is definitely a feasible and critical task, especially at this time with the increasing input of new viral genomes from virome studies conducted in a variety of previously unexplored biomes. In a future update, a new set of ViPhOGs can be seamlessly integrated into VIRify.

A second concern related to the taxonomic assignments, is that this pipeline does not seem to have been tested or validated using existing complete virus genomes, only using mock community assemblies which could have many incomplete genome fragments (lines 360-363). In my opinion, it is important to understand how accurate the taxonomic predictions are in the most ideal situation, in order to understand the limitations of the pipeline.

Thank you for pointing this out. We agree that using complete viral genomes with known taxonomy for testing and validation would have been an adequate and sensible approach to benchmark our pipeline. We considered following this approach during the early stages of pipeline development, but we decided to use mock community datasets instead to benchmark our pipeline. One of the reasons for making this decision was that we wanted to conduct the tests using datasets that reflect more closely the data that we expect the community to analyze with our pipeline. Using the mock community datasets allowed us to assess the pipeline's performance on nearly-complete genomes (that we obtained in both mock community assemblies), genome fragments, and in the presence of contaminant sequences. Another reason for using mock community datasets instead of complete reference genomes is that the latter were used for calculating taxon-specific taxonomic assignment parameters. In particular, for this revision of our work, we calculated taxon-specific cut-offs for taxonomy assignment using all the complete reference viral genomes available in the NCBI databases. Therefore, we were concerned that using these genomes for benchmarking could have led to a bias in our testing and validation results, in turn providing an imprecise picture of the pipeline's performance for real-case scenarios.

As part of this review, I have installed and run the pipeline on some test data and will be providing observations and some recommendations.

Thank you for making the effort and testing the pipeline and the code itself so carefully!

Installation: I used an ubuntu virtual machine and used the nextflow install as was suggested on the GitHub page. Installation was seamless and quick. I tried a similar installation locally on a Mac using the terminal and ran into versioning issues with nextflow and the virify pipeline and abandoned this route.

We're sorry that the installation via Nextflow did not run directly on Mac. We're using the pipeline on both Linux machines (laptop, cluster) and Macs, which is generally working. Sometimes, on Mac, there are problems with the installed Java Runtime that Nextflow needs.

What you can try: installing Nextflow via Conda and all necessary dependencies and then installing and running VIRify from that environment:

conda create -n nextflow -c bioconda nextflow
conda activate nextflow
nextflow pull EBI-Metagenomics/emg-viral-pipeline
nextflow info EBI-Metagenomics/emg-viral-pipeline

If this does still not work, please feel free to report an https://github.com/EBI-Metagenomics/emg-viral-pipeline/issues.

Running pipeline: I used a mock dataset of 5 complete bacteriophage genomes which were all similar to well-characterised phages but not necessarily identical. The pipeline ran very quickly and easily. I ran a second set of ~5000 contigs from a virome which finished overnight. No issues.

We are pleased that it worked out and that our efforts to build a solid pipeline were not in vain.

Results of pipeline:
Small dataset:
Four out of the five bacteriophages, including the reference RNA phage MS2 were categorised as low confidence predictions in the first step of the pipeline. For me, this calls into question the thresholds that were used to categorise high confidence versus low confidence. Could the authors benchmark the virus detection categorisation with a set of known viruses from GenBank to see if the distinction between high-confidence and low-confidence needs to be made and what the best thresholds should be?

Thank you for pointing this out. The parameters we selected for the viral prediction part are based on previous studies that employed a combination of VirSorter and VirFinder for predicting viral sequences in metagenomic assemblies from both marine and human gut samples (references 14 and 36 in manuscript). One of the main reasons we had for splitting the predictions into high and low confidence is that, based on previous reports (reference 79 in manuscript) and our own experience, both VirFinder and PPR-Meta are more prone to reporting false positives than VirSorter. On the other hand, we are aware that our current categorization likely favors the presence of sequences from dsDNA phages in the high confidence category, as this type of viruses is the one mainly targeted by VirSorter. To improve VirSorter's performance, we included the --virome option in our pipeline, which is more suitable for analyzing virome datasets and for covering a wider range of target viruses. Despite the current categorization, we advise the users to follow the default pipeline's approach, which is to annotate the full set of viral predictions from HC and LC categories, as this most likely increases the detection of viral lineages that are less represented in the public databases.

Taken into account the outdated taxonomy database used, the taxonomic assignments of the phages were correct at the genus level for only 2 out of 5 phages. MS2 was correctly classified at the family level but no genus level classification. One phage was correctly assigned at the order level and one was unclassified. No incorrect assignments were made. However, low

sensitivity at the genus and family level means that there is some optimisation required for the "voting system" or potentially an update to the ViPhOG database itself to include a more up-to-date set. For the smaller viruses, a threshold for at least two hits at the genus level (lines 352-353) may be too stringent. For example, most members of the phylum Cressdnaviricota only encode two proteins.

Thanks a lot for this detailed test. As detailed in the latest version of the manuscript, we modified the taxonomic assignment algorithm to incorporate a set of taxon-specific thresholds that were calculated using the complete reference viral genomes available in NCBI databases. These new thresholds take into account the number of assigned informative ViPhOGs and the average number of CDS among the genomes that comprise each taxon. As you suggested, this modification was particularly advantageous for viral taxa characterized by small genomes and that had rather few informative ViPhOGs assigned to them. These modifications increased the number of correctly classified contigs in both of the tested mock communities, with a minor increase in false positive assignments. Among these correctly classified contigs were short sequences from phage phiX174, Rotavirus A and Bovine herpesvirus 1. We also agree that a current limitation in any predictor is the training set, and with the current amount of sequences obtaining high accuracy at lower taxonomic levels (genus and species) is challenging. As more viral genomes become available from different species within each genera, better training sets could be developed.

Larger dataset:
Of the ~5000 contigs that I considered potentially viral based on an internal dataset using VirSorter2 and VIBRANT, 921 were considered viral using VIRify. The decision to minimise false positives therefore may have a consequences for an increased amount of false negatives. I consider this a feature, rather than a bug, because it's a decision that needs to be made in viromics research. However, I do suggest that the authors be clear about this in the manuscript and pipeline.

Thanks for your suggestion. As you have pointed out, while we were designing VIRify we decided to favour specificity over sensitivity. We thought that this would be a more convenient approach to follow in order to develop a resource that provides trustworthy results, and that could be applied to any type of sample. However, we also provide the option '--onlyannotate' that allows users to skip the viral prediction step and apply the taxonomic annotation pipeline to a complete set of input contigs. If users follow this approach, they should be aware of longer run times, more resource consumption and false positive viral predictions. We added more information in the manuscript to further clarify these points.

The taxonomic assignments that were made were largely unclassified viruses (780) and the rest of them were assigned to the order Caudovirales. The family, subfamily and genus assignments appeared to be correct.

As explained above, due to the changes we made to the pipeline, there should now be fewer unclassified viruses.

Overall, I found the pipeline easy to work with and its outputs easy to understand and use. My assessment is that it favours specificity over sensitivity in both the virus detection and taxonomic assignments.

Thanks, we're very happy about this positive feedback and fully agree: VIRify and the implemented approach is a rather conservative pipeline. However, we also see this as a feature to reduce false positive taxonomic assignments from accumulating in public databases.

In terms of the manuscript, my final comment is that the literature/introduction and discussion should be updated to account for pipelines and new tools that have been developed. The authors have already indicated that they may update the tool VirSorter to VirSorter2, but there is also an update of VirFinder to DeepVirFinder and new tools such as VIBRANT that have been developed since the authors started working on VIRify. Similarly, other pipelines have been published, for example MetaPhage and Hecatomb, and potentially others. I do not expect these to be benchmarked but they should at least be acknowledged in the introduction or discussion.

Thanks for the suggestion. Both VIBRANT and DeepVirFinder were included in our benchmarking of viral prediction tools. We updated the manuscript to acknowledge recent pipelines in the introduction (lines 144 - 147).
